# Designing and delivering bioinformatics project-based learning in East Africa

**DOI:** 10.1186/s12859-024-05680-2

**Published:** 2024-04-14

**Authors:** Caleb K. Kibet, Jean-Baka Domelevo Entfellner, Daudi Jjingo, Etienne Pierre de Villiers, Santie de Villiers, Karen Wambui, Sam Kinyanjui, Daniel Masiga

**Affiliations:** 1 International Centre of Insect Physiology and Ecology (ICIPE), P.O. Box 30772, Nairobi, 00100 Kenya; 2 International Livestock Research Institute, Nairobi, Kenya; 3 Department of Computer Science, Makerere University, P.O. Box 7062, Kampala, Uganda; 4 KEMRI-WellcomeTrust Research Programme, P.O. Box 230-80108, Kilifi, Kenya; 5 Pwani University, Mombasa -Malindi Highway, P.O. Box 195-80108, Kilifi, Kenya; 6 African Center of Excellence in Bioinformatics, Makerere University, P.O. Box 7062, Kampala, Uganda; 7 Nuffield Department of Medicine, Oxford University, Oxford, UK

**Keywords:** Project-Based Learning, Bioinformatics, East Africa, EANBiT, H3Africa, Capacity building

## Abstract

**Background:**

The Eastern Africa Network for Bioinformatics Training (EANBiT) has matured through continuous evaluation, feedback, and codesign. We highlight how the program has evolved to meet challenges and achieve its goals and how experiential learning through mini projects enhances the acquisition of skills and collaboration. We continued to learn and grow through honest feedback and evaluation of the program, trainers, and modules, enabling us to provide robust training even during the Coronavirus disease 2019 (COVID-19) pandemic, when we had to redesign the program due to restricted travel and in person group meetings.

**Results:**

In response to the pandemic, we developed a program to maintain “residential” training experiences and benefits remotely. We had to answer the following questions: What must change to still achieve the RT goals? What optimal platforms should be used? How would we manage connectivity and data challenges? How could we avoid online fatigue? Going virtual presented an opportunity to reflect on the essence and uniqueness of the program and its ability to meet the objective of strengthening bioinformatics skills among the cohorts of students using different delivery approaches. It allowed an increase in the number of participants. Evaluating each program component is critical for improvement, primarily when feedback feeds into the program's continuous amendment. Initially, the participants noted that there were too many modules, insufficient time, and a lack of hands-on training as a result of too much focus on theory. In the subsequent iterations, we reduced the number of modules from 27 to five, created a harmonized repository for the materials on GitHub, and introduced project-based learning through the mini projects.

**Conclusion:**

We demonstrate that implementing a program design through detailed monitoring and evaluation leads to success, especially when participants who are the best fit for the program are selected on an appropriate level of skills, motivation, and commitment.

**Supplementary Information:**

The online version contains supplementary material available at 10.1186/s12859-024-05680-2.

## Background

The Eastern Africa Network for Bioinformatics Training is a collaboration between universities (Makerere in Uganda and Pwani in Kenya) and five research institutes in Kenya and Uganda. EANBiT offers a two-year Master of Science (MSc) in Bioinformatics training and research project placement. Since 2018, EANBiT has hosted a 5-week residential training (RT) as an integral component of the MSc in Bioinformatics programs at Pwani University in Kenya and Makerere University in Uganda, which it helped establish. The RT aims to strengthen bioinformatics skills among the cohorts of students who have completed the coursework in preparation for research project placement. EANBiT is part of the Human Heredity and Health in Africa Consortium (H3Africa) training program to develop bioinformatics and genomics expertise in Africa through postgraduate training to support the capacity building for the analysis of genomic data generated by the consortium and other programs [1].

A critical skill shortage in Africa to support genomic data analysis is widely acknowledged [2–6]. The H3Africa Bioinformatics Network (H3ABioNet) and similar programs address this shortage through short-term training [1, 7] including an introduction to bioinformatics training, which uses a blended learning approach [8]. However, these efforts provide only narrow niche skills. They cannot generate a pool of genomic experts to lead research efforts [1, 3], as they run over a short time.

Developing a critical mass of research leaders on any subject requires a large starting pool of high-quality trainees [2]. Standard masters programs immerse students in theoretical concepts with practical exposure and project-based learning during research work [9]. Some masters programs, especially in Africa, are knowledge-oriented and exam-oriented and do not offer the depth of technical expertise needed to prepare students for research projects [10, 11]. Short-term training (less than a week) can introduce only a few concepts [12]. Therefore, there is a need for much longer training programs, boot camps, summer training, or RT that focuses on crucial skills [7]. Therefore, as a component of MSc programs at partner universities, we introduced a RT program that fills technical skill gaps to prepare for the project phase after coursework and exposes them to a collaborative research environment through group projects with the soft skills needed to succeed. The RT does not form part of the credit system at Pwani University and Makerere University.

EANBiT-RT has matured through continuous evaluation, feedback, and codesign over the four years. However, 2020 challenged and tested the program due to the COVID-19 pandemic, which restricted in-person travel and group activities. We could not offer EANBiT-RT in person, a significant change in the design and implementation of the program. Since trainees and trainers could not converge on site, virtual training was the only option. There are three main learning modes: face-to-face, hybrid, and online; each form has strengths and weaknesses [13]. Face-to-face is the traditional form that has matured through years of pedagogical research, with individualized touch, close interaction between trainers and trainees, and immediate feedback. EANBiT-RT initially adopted face-to-face to immerse students in a collaborative and peer learning environment. Online learning increases access to training and is cost-effective, but Internet connectivity remains a significant challenge, especially in Africa [8]. The hybrid approach model used by H3ABioNet, a blended multi-delivery mode learning approach, combines the best of both worlds [5, 14].

## Methods

### EANBiT-RT design and implementation

#### 2018: ‘train the trainer'

The first iteration of EANBiT-RT was hosted at multiple sites sequentially from 11 June to 13 July 2018: International Centre of Insect Physiology and Ecology in Nairobi, the KEMRI Welcome Trust Research Program (KWTRP), and Pwani University in Kilifi. The "train the trainers" RT aimed to strengthen the technical capacity in bioinformatics of individuals working in collaborating EANBiT universities and research institutes as project supervisors. The 2018 RT program started with an introduction to bioinformatics, molecular biology programming, NGS bioinformatics, population genetics and soft skills, including scientific writing, ethics, and open science (Table 1). Under this umbrella, 27 topics were taught by 25 trainers who decided on the course content and the mode of delivery. This approach did not provide the best learning outcomes for the training participants but ensured a broad introduction to the topics.

**Table 1:** 2018 EANBiT-RT program

Date	Course
11–15 June 2018 (Week 01)	*Introduction lectures on bioinformatics *Introduction lectures on molecular biology *Introduction Lectures on Computing for Biology *Presentation of the recent history (since Sanger Sequencing) of sequencing technologies
18 July–06 July 2018 (Week 02–04)	*Basic experimental design, biostatistics, manipulation of tabular data in R, and basic visualization (Scatter plots, box plots, regression lines) *Sequence alignments, BLAST tools, motifs, homology modeling (HMMs), profile databases (Pfam, Prosite, etc.) *Introduction to NGS data (Illumina short reads technology), their vocabulary (coverage, clusters, reads, etc.), and their different uses: RNAseq, whole genome assembly, amplicon sequencing *Whole genome assembly (de novo and reference-based) *Genome annotation * Barcodes, markers, and 16S rRNA analysis, Metagenomics/Microbiomes *Molecular evolution and phylogenetics, selection pressure (dN/dS analyses) *Population structure, Population genetics *Polymorphisms, variant calls, and genome-wide analysis studies (GWAS) *Genomic selection, marker-assisted selection *Transcriptomics *Protein structure, folding *Advanced UNIX command line (Bash scripting, sed, awk, etc.) *Advanced Python *Detailed analysis of Oxford Nanopore data sets
09–13 July 2018 (Week 05)	*Scientific writing (papers and proposals) Scientific communication (to an audience of non-specialists) *Grant writing *Project management (Gantt charts, monitoring, evaluation, etc.) *Pedagogy *Academic integrity, authorship, collaboration management (MoU), supervision, leadership skills, and conflict resolution * Research ethics (responsible conduct, research on human subjects, etc.) *Literature management (references, online journal databases) and paper critique *Open Science, Issues of Reproducibility, Open Source Data, and Publications

Although well received, the key weakness of this iteration was the inadequate hands-on components due to the limited time available for each module. We learned a lot from these weaknesses through feedback received from participants and trainers. Despite the weaknesses, the continuous evaluation and feedback of the program allowed us to learn and adapt: a success of the pilot.

The trainers came from both Africa and Europe: Kenya (14), Uganda (4), Tunisia (4), France (2), the United Kingdom (1), South Africa (1), and Sweden (1). They taught various modules according to their preferences and areas of expertise. Although originally designed to be delivered sequentially, the availability of trainers changed the course order. Since the trainers could not coordinate to harmonize the content, there were overlaps and omissions, compounded by the considerable number of modules and trainers, which limited the time allocated for each course and made RT more theoretical, with limited opportunity for practical skills training. The main recommendations of the evaluation were the need for practical training with adequate time allocation, including central organization of the course materials. However, the students praised the program organization, especially for hosting training in two venues (Nairobi and Kilifi), which helped to minimize locational monotony.

#### 2019: redesign of the program

The second year of the EANBiT-RT was significantly different from the first year. The Curriculum Development and Implementation Workgroup (CDIwg) designed the program, drawing lessons and recommendations from feedback and evaluations from the first year to design a program that meets the objective of EANBiT RT (Table 2). For this second iteration, our focus shifted to participants who had completed their first year of MSc in bioinformatics coursework and already had a background in bioinformatics and programming. What skills would they need for their projects, and what skills gaps in the course work could the RT fill? We needed to immerse students in a bioinformatics research environment through practical modules, joint mini projects, and daily coding exercises. This approach, called experiential or active learning, involves learning by doing and is ideal for the diverse participants [15] attracted to EANBiT-RT to instill collaboration and improve learning outcomes and confidence in applying knowledge in the real world. Our program design was also informed by the need to instill open, reproducible, collaborative research practices and open science in our trainees. Finally, learning from the disconnected training in the previous year, we created a GitHub repository where all trainers could host their training materials and collaborate on module design and delivery.

**Table 2:** 2019 EANBiT-RT program designed with four tracks: technical training, seminars, daily coding, and mini projects. The key modules are in bold

Week	Course	Allocated (HRS)
1	Technical: Version control and collaborative development (Git & GitHub & Slack)	6
	Technical: Advanced Scripting	5
	Coding: Linux command line	1
	Technical: Gene models and annotation	6
	Coding: HPC SSH (SLURM scheduler)	1
	Technical: Biological databases and API	2
	Technical: Submission to public databases	2
	Project: Presentation of mini projects	2.5
	Assign groups	1
	Coding: Bash (sed, Awk, Regex)	1
	Soft skills: Scientific Writing and Presentation Skills	3
	Project: Review of mini project methods	Weekend
2	Technical: Reproducibility: package management and workflow languages (Nextflow, Snakemake)	3
	Coding: Python	1
	Coding: Jupyter Notebooks	1
	Technical: Specialized databases (VectorBase, EupathDB) and APIs	1
	Technical: Practical proteomics (domain databases, GO)	2
	Technical: Pathways, Gene Regulation Networks (KEGG)	2
	Project: Develop a mini project methodology	1
	Meetings with mentors	1
	Technical: Phylogenomic (Visualization and Annotation)	3
	Project: Present mini project group concepts	0.5
	Seminars: Human health	1
	Project: Research Consultations	1
3	Technical: Metagenomics (16S and whole genome shotgun sequencing)	3
	Coding: Practice R	1
	Coding: R-markdown	1
	Technical: Machine Learning and Modeling for Big Data (fuzzy strings, HMM)	5
	Coding: Shiny Apps	1
	Project: Meetings with mentors	1
	Seminar: Marine Sector	1
	Project: Implementation of mini projects	7
4	Project: Implementation of mini projects	7
	Seminar: Agricultural sector	1
	Project: Meetings with mentors	1
	Soft skills: Introduction to the Purpose Roadmap-PRM	2
5	EU PATH DB TRAINING	
	Project: Implementation of mini projects	Four days
6	Project: Presentations of mini project reports	One day
	Project: M.Sc. project presentations	Throughout the week
	Soft skills: Student presentations on PRM	2

The second year RT learned from the evaluation of year 1. We developed learning objectives and outcomes and mapped the program to ISCB bioinformatics competencies [16, 17] to fill the training gap after coursework and enable them to apply the skills according to Bloom's taxonomy [18] (Table 3). We wanted to produce all-round bioinformatics trainees through soft skills training in scientific writing and a purpose roadmap, with a key emphasis on mentorship. The program had four tracks: technical training focused bioinformatics and genomics, seminars for talks around various themes, daily coding exercises to sharpen programming skills, and projects. The technical training track had 12 modules to cover in three weeks. The same year, participants benefited from a week-long VEuPathDB training in *icipe*. Although there was a significant improvement over the previous year, the trainers could not comprehensively cover all the modules.

**Table 3:** Mapping of EANBiT-RT modules to ISCB competencies and Blooms

Module	ISCB competency	Blooms taxonomy
Technical: Version control and collaborative development (Git & GitHub & Slack)	F: Bioinformatics tools and their use J: Scripting and programming appropriate to the discipline	Application
Technical: Advanced scripting (including SED, AWK, and REGX)	F: Bioinformatics tools and their use G: Fundamentals of Computer Science Systems J: Scripting and programming appropriate to the discipline	Application
Technical: Advanced R, tidyverse, R- markdown, Shiny apps (with practical)	F: Bioinformatics tools and their use J: Scripting and programming appropriate to the discipline	Application
Technical: Reproducibility (incl. notebooks) and package management: workflow languages (Nextflow, Snakemake) and containerization (Docker and Singularity)	H: Computing requirements appropriate to solve a given scientific problem J: Scripting and programming appropriate to the discipline K: Construction of software systems of varying complexity based on design and development principles	Knowledge Application
Technical: Whole genome genomics: Assembly, Metagenomics	C: Biological data generation technologies F: Bioinformatics tools and their use	Knowledge Application
Technical: Long-read sequencing and its applications, with data analysis	C: Biological data generation technologies F: Bioinformatics tools and their use	Knowledge Application
Project: Mini projects	O: Effective teamwork to achieve a common scientific goal G: The ability of a computer-based system, process, algorithm, component, or program to meet desired needs in scientific environments/problems	Application
Soft skills and Seminars: Ethics, Scientific communication, open science	L: Local and global impact of bioinformatics and genomics on individuals, organizations, and society N: Effective communication of bioinformatics and genomics problems, issues, and topics with various audiences, including, but not limited to, other bioinformatics professionals M: Professional, ethical, legal, security, and social issues and responsibilities of bioinformatics and genomics Data in the workplace	Knowledge Synthesis to Evaluation Knowledge

#### 2020: going virtual

In February 2020, the CDIwg met and designed an improved program, with feedback from previous years, which changed when we could not offer the training in person: we had to redesign a virtual program. The team met again in March 2020 to redesign the program. The redesign adjusted the program to focus only on some key modules, make the training eight weeks long to avoid online fatigue from long hours and agree on the technology to use (Table 4). The training was only done in the morning, with the afternoon reserved for group work, practical or presentations. We selected key modules around reproducibility (GitHub, workflows, and containers) and Next-Generation Sequencing (whole genome, metagenomics, long-read sequencing). The team also raised three key training requirements for moving online: video conferencing, collaboration, and resource sharing.

**Table 4:** EANBiT-RT program for 2020, 2021 and 2022

Week	Modules	Day and Date
1	Technical: Version control and collaborative development (Git & GitHub & Slack)	6 July
	Technical: Reproducibility and package management: workflow languages (Nextflow, Snakemake) and containerization (Docker and Singularity)	8–10 July
	Project: Presentation of mini projects by the mentors and assign groups	10 July
2	ISMB Conference	13 to 17 July
3	Technical: Advanced scripting (including SED, AWK, and REGX)	20–21 July
	Soft skills: Introduction to the Purpose Roadmap—PRM	22 July
	Technical: Advanced R, tidyverse, R- markdown, Shiny apps (with practical)	23–24 July
	Project: Present mini project group concepts (how they plan to tackle them)	24 July Afternoon
4	Soft skills: Scientific writing and presentation skills	27 July
	Technical: Whole genome genomics: Metagenomics (16S and whole genome shotgun sequencing) + reference mapping + RNA sequence	28–31 July
	Seminars: Open Science	29 July
	Project: Research Consultation	31 July
5	Technical: Long-read sequencing and its applications, with data analysis	3 August–5 August
	Project: Implementation of mini projects	6 August
	Soft skills: Peer Coaching for Personal Career Development	7 August
	Project: Research Consultation	7 August
6	Project: Implementation of mini projects	10–11 August
	Seminar: Bioinformatics and Society Benefits	11 August
	Seminar: Privacy, Ethics, and Data Protection in Health Research and Data	12 August
	Soft Skills: Work Ethics	13 August
	Project: Implementation of mini projects	14 August
	Project: Research Consultation	14 August
7	Project: Implementation of mini projects	17–18 August
	Soft skills: Feedback on the Purpose Road Map—PRM	19 August
	Project: Implementation of mini projects	August 20–21
	Project: Research Consultation	21 August
8	Project: Implementation of mini projects	24–27 August
	Project: Final presentation	28 August

The same program was used in subsequent offerings, with only dates changing

The EANBiT’s curriculum development and training working group reviewed all standard tools. They proposed the most preferred tool or platform based on the features used by the various user groups, i.e., trainers and students, and the desire to overcome online training challenges. Other options that we considered are highlighted in Additional file 1. For the 2020 EANBiT virtual training, we decided on a combination of tools. Zoom for live training [19, 20], GitHub for content management and collaboration, and Slack [21, 22] for discussions, Q & A, and long-term interactions. All sessions were recorded and shared privately and securely with users through the cloud. Therefore, participants who drop out due to internet or power issues could catch up on the training.

In addition, teaching assistants could help those with technical difficulties using breakout rooms, especially for installation debugging. Without in-person interaction for quick communications, we needed a platform that allowed organizers to share announcements, trainers to interact with participants and share links, and participants to interact. Students spend considerable time on mini projects. Therefore, we could create separate Slack channels to share ideas, tools, and files. We created a channel for each module: *genomics, reproducibility, Linux command line, advanced r, seminars, and soft skills.* We also had channels for *announcements, recordings, mini projects, feedback*, a private channel for the trainers, and each mini project group. We used collaborative notes to share details to access recordings and keep track of attendance. Finally, we created the EANBiT-RT GitHub organization to communicate course material and content and manage mini projects, where each group had a repository. Using GitHub enabled project team members to collaborate on a mini project, with seamless code reviews by multiple team members working on a single project.

#### 2021 and 2022: hybrid approach

Initial iterations, program goals, trainee composition, and, recently, delivery mode and feedback have continued to shape the design of the RT through subsequent offerings. The mature program used in 2021 and 2022 is the same as the virtual program (Table 4). However, it ran for 5 weeks, with the first three weeks of taught modules offered virtually, while the last two weeks of the project phase offered in person. It used practical technical training, soft skills, seminars, and collaborative mini projects. Each component of the program aimed to fill a skill gap in reproducibility and genomics, prepare students for research projects, and advance their bioinformatics careers. As the program matured, it also became cost-efficient, recognizing the in person mini project phase as the most important component of implementing a blended approach without losing quality.

### Participants selection process

**Fig. 1:**
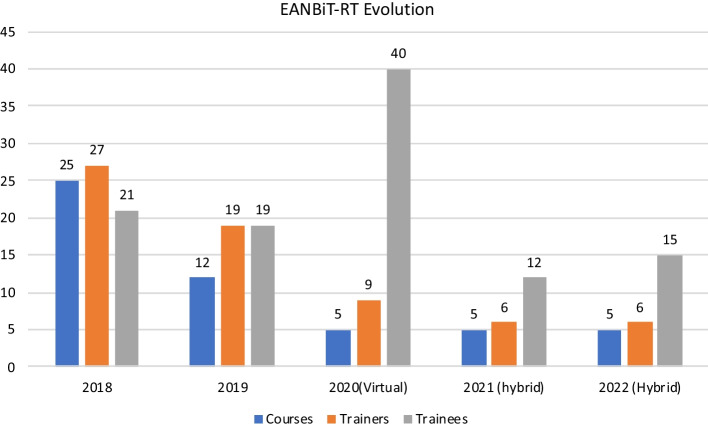
Evolution of the program as observed by the number of modules/courses, trainers, and trainees

The first iteration made open calls for applications since the MSc programs at Pwani and Makerere University had not been established. Three reviewers reviewed each application: the EANBiT Steering Committee and Curriculum Development and Implementation Work Group members. For this train-the-trainer phase, those selected were lecturers, Ph.D. and MSc students. In year 2, we closed the call to the EANBiT partner institutions, selecting M.Sc. or Ph.D. students at the same level as Makerere and Pwani University students. From year three onward, the program was limited to participants undertaking M.Sc. at Pwani University and Makerere University; all available and interested students attended the training (Fig. 1).

#### Mini projects

The trainers designed mini projects around the modules to ensure the participants could gain a sufficiently deep understanding and apply the skills gained (Table 5). The projects are based on ongoing work by the mentors and collaborators or on reproducing a published research article. The project concepts are posted to the GitHub repository, and participants are asked to choose at least two projects by responding to the GitHub issue in order of preference. In consultations with the training coordinator, 4–6 students are assigned to each project using GitHub issues. We created a private GitHub repository for each project and added participants as collaborators. Participants are expected to document all their code and, where possible, convert it to a workflow language and use containers. Participants are encouraged to use GitHub projects to manage their work and to employ best practices in Git collaborations. At the end of the second week, participants present how they planned to tackle the project, collaborate, and document their work. Throughout the implementation of the project, the mentors are available in person and/or virtually for consultation. A mentor was always available in the training room (physical or Virtual); the project leads scheduled consultation sessions with the project mentors.

**Table 5:** Mini projects designed for EANBiT-RT

Cohort	Mini project topic
5	NextFlow pipeline for ONT long read meta-transcriptomic data analysis
	Stingless Bees Metabarcoding
	ONT Whole Genome Assembly—*E. Coli*
	Sea turtle immunogenetics
	Antelope phylogenetics
	Chelonid Alphaherpervirus 5 phylogeny
4	Whole Genome Assembly and Annotation of SARS-CoV2
	Machine Learning for Genomics
	ONT Metatranscriptomics Mini project
3	Reproducing a Machine Learning paper
	Reproduce published ONT pipeline
	Genomic Characterization of E-coli Isolates from Rural Drinking Water Systems
	Interrogating Public Data Sets and Paths to Results on Covid19 in East Africa
	Origin of antimicrobial resistance E-Coli isolates in a pastoralist community using whole genome sequence data
	Develop: RNA-Seq data processing and gene expression analysis workflow
	Variant call from NGS data of two accessions of Lablab purpureus
2	Microbiome of Seagrass species on the Kenyan coast
	Microbiome Metagenomics Analysis of Kilifi Creek Mangrove Sediments
	Reproduce: Metagenomic analysis of viruses associated with maize lethal necrosis in Kenya
	Develop a variant call-to-action workflow pipeline
	Whole Genome Assembly, Annotation, and Phylogenetic Analysis of Rice Fungus

#### Monitoring and evaluation

The program included continuous evaluation and feedback for all the iterations. Students evaluate the training through anonymous feedback forms administered before, weekly, and after training (Additional files 2, 3 and 4). Before training, we assessed the level of skills and expectations of participants; during training, we assessed their satisfaction; and at the end of the training, we assessed their skills again to determine improvement and seek feedback. After training, the organizers spent time with the participants to reflect on the implementation of the program and receive oral feedback.

## Results

We describe a Project-based and hands-on learning implementation for EANBiT, a critical component in preparing students for research. The students are better prepared for research by offering RT as a bridge between the course and project work. EANBiT-RT delivery and implementation has evolved to meet the needs and adapt to emerging challenges, such as COVID-19. We also discuss how monitoring and evaluation have been critical to the project's success, the role of mini projects in enhanced learning and make some recommendations for adopting project-based learning.

### Monitoring and evaluation are critical to the maturity of the program

**Fig. 2:**
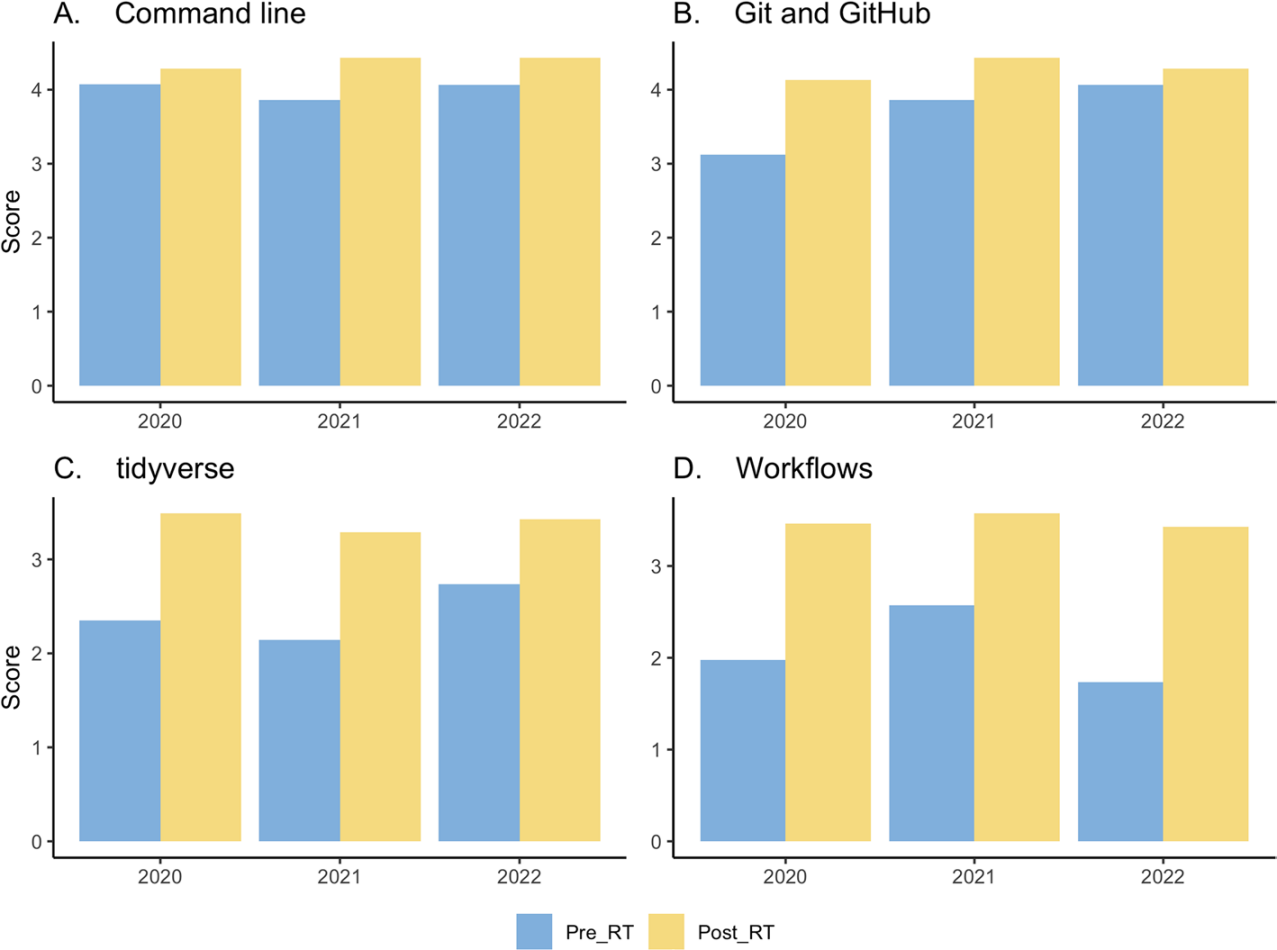
Improvement after residential training in Command line, Git and GitHub, tidyverse, and workflow languages. The score is based on a rating of how comfortable they were with the topic/tool between 0 (not at all) and 5 (Very comfortable); the number of responses is 40, 12 and 15 for 2020, 2021 and 2022, respectively

The design and delivery of RT evolved and matured through feedback from students and trainers. We note the need to evaluate each stage of program design and implementation. It is easy to overlook the process of selecting participants or trainers and monitoring the implementation of the program as designed. Post-workshop surveys revealed a marked improvement in proficiency and confidence in applying technical concepts (Fig. 2).

Numerical feedback does not tell the entire story or provide useful feedback: topics covered relevance (100% positive), use of the skills gained (84% strongly agree), trainers' understanding of content (84.2% strongly agree, 15.8% partially agreed) point to the successful 2018 iteration. However, the question that sought recommendations from participants revealed the weaknesses of the iteration, supporting the observation that the numerical rating can be misleading or have a negative impact, especially without a recommendation [15, 23]. The question “What aspects of the training did you like and benefit from the most?” highlights the program's critical components: trainers, training approach, or content. The best feedback leads to change for improvement; otherwise, it will be simply a punitive exercise and nothing different from the guesswork of designing without evaluation [23].

### Virtual learning can be hands-on

Virtual training differs from in-person meetings. With a mature RT program, CDIwg (Curriculum Development and Implementation workgroup) discussed how to replicate the RT experience virtually. It had to answer the following questions: What should change to achieve RT goals, what optimal platforms are available to use, how to manage connectivity and data challenges, and how to avoid online fatigue? Narrowing down these critical questions, the team refined the design focusing on what makes training unique and successful. Reduced the training modules to five and spread the training over eight weeks to allow enough time to learn and avoid 'zoom fatigue.' We further reduced the seminars from the original 6 to two, hosted as panel discussions, bioinformatics careers through EANBiT independent Scientific Advisory Board (*i*SAB) and Open Science. As the last component, we retained three lead trainers, also members of the CDIwg, responsible for coordinating the technical modules, including sourcing additional trainers. Participants were supported with cash to purchase mobile internet bundles, while recorded sessions allowed those who lost connectivity to catch up on course content.

Remote practical training requires competency in the collaborative tools chosen for the program. Therefore, the first week of training aimed to impart reproducible and collaborative research tools, including Git and GitHub, workflow languages, and containers. The evaluations showed that “practical learning was highly informative” and that “the mini project was also a highlight, especially the collaboration with students from other institutions.” Another noted that they learned the most from the mini project part of the training, first because of that experience working collaboratively remotely with other members during the projects and incorporating the tools and soft skills taught during the training to solve real problems. Although online, we could provide an experiential learning experience for our students due to design, tools, and closely monitored implementation. A member of the CDIwg was present throughout the training, along with a technical support team available via Slack.

### Mini projects are an integral part of enhanced learning

The mini projects designed for the program, hosted on GitHub Repo, were well received as highlights of the RT. They were designed to allow trainees to use all their learned skills. They required them to work closely using people skills in groups of 4–6 individuals. Groups work together using collaborative tools, present their findings using communication skills, and analyze data using technical bioinformatics skills. It allowed them to strengthen their concepts. The importance of the mini projects was a recurring theme in the feedback: “… I also enjoyed working on the mini project; it was a new collaboration experience and worked flawlessly. I learned many things working with long reads and the tools used for long reads: the hands-on aspect and the mini project enhanced their understanding.” The participants mentioned Mini projects on 11/40, 2020, 6/12 for 2022, and 10/15 for 2022 way more than any other training components. Participants chose projects based on interest and skills and thus could fully engage.

Some teams worked on a manuscript and a further analysis for the project after the RT. These observations agree with the literature that project-based learning allows learners to drive learning, collaborate, and impart soft skills such as problem-solving, critical thinking, and time management [12, 24].

### Challenges and opportunities

**Fig. 3:**
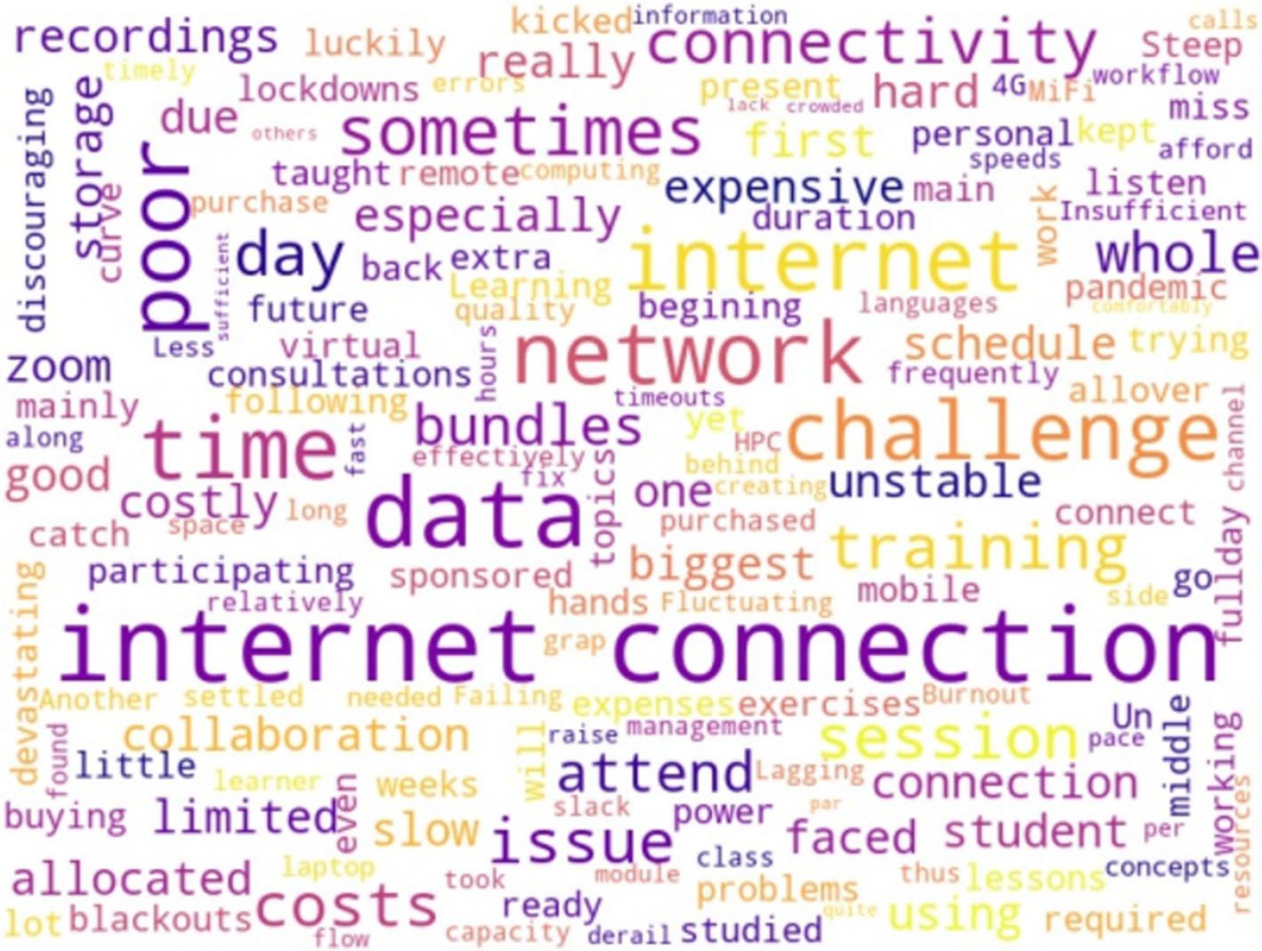
Major challenges in the word cloud gathered from 2020 fully virtual training. The word cloud was generated from 39 responses to the question, “What are some challenges that you faced while participating in the training?” Internet and connection issues were the main barriers to the smooth running of virtual training

The EANBiT RT course is designed to be immersive with a standardized training environment, direct contact with the trainers, and access to high-speed internet and computer infrastructure. The main challenges highlighted by the trainees were the cost of internet access, the long training hours, and the lack of personal experience (Fig. 3). “Sometimes, the network connectivity was down, and trying to reconnect and catch up, especially during hands-on exercises, was devastating and discouraging. However, I followed the Zoom recordings after the session.” Others observed that network issues would derail the flow of information. Then the pace would sometimes be too fast. You cannot discuss an issue comfortably and be on the same page as others. Some trainers also found the training to be tiring and the pace slow.

Despite the challenges, we have met our goals with RT through the completely virtual training in 2020 and subsequent hybrid RT. As a participant noted, “the training shaped my research approach and exposed me to more tools and solutions to problems that I had faced for a long time in bioinformatics.” Others found the training eye-opening by shedding light on the career paths and expectations of a bioinformatician. Participants left the training knowing how to chart their careers in bioinformatics, how to collaborate effectively, and the technical skills to tackle genomic research projects. Hosting the training virtually had some opportunities. We were able to train a much larger number of participants. As one participant stated, “I am only grateful to the organizers of this training for allowing us to train with them virtually in the middle of a pandemic. Initially, I could not attend this training if everything remained normal (no lockdowns). I have learned a lot!”.

Additionally, hosting the RT virtually allowed us to incorporate the ISMB (Intelligent Systems for Molecular Biology) conference, also held online, into the program. EANBiT paid the registration fee for EANBiT fellows to attend and prepare a presentation to the rest of the participants on lessons learned and experience from the tracks they attended. This further gave them conference exposure and experience to network and learn new research in computational biology. This was possible due to cost savings from hosting training remotely.

## Discussion

Residential training is integral to the MSc programs at EANBiT partner universities. It offers the participants an opportunity to acquire practical and real-world skills necessary to execute the MSc research and prepare them for a career in bioinformatics. We have experienced tremendous growth in the participants after the program. Participants maintained a close relationship with EANBiT, some of whom taught students at partner universities, including returning to RT as trainers, demonstrating the quality of training they received. We continued to learn and grow through evaluating the program, the trainers, and the modules, enabling us to provide robust training during a pandemic. Evaluating each program component is critical for improvement, primarily when feedback feeds the program's improved design. The Pandemic challenge presented an opportunity to reflect on the essence of the program, what makes it unique, and to focus on delivering on its objective: strengthening bioinformatics skills among the cohorts of students who come through the program.

It takes a village to raise a child [25]. Through RT, we strengthened our working relationships with BReCA (https://breca.mak.ac.ug/) and H3ABioNet, and H3Africa to support the program as trainers, speakers, and as part of panels. We have achieved a good balance between local and guest trainers, including bringing former participants on board to assist with the training. A well-designed program is not useful when it is not closely monitored and implemented. The working group involved in the design should be involved in the implementation. Virtual training was successful due to close monitoring of the implantation design, the participants' attendance, timely sourcing of the mini projects from the trainers, and close supervision of their implementation. The trainers supervised the mini projects, allocated compute resources required through their system administrators, and answered the questions from the participants on Slack.

A program is designed with a particular group of participants [26]. Therefore, a successful program must select the best fit for the program: an appropriate level of skills, motivation, and commitment [10]. Since we offered training to all students in the bioinformatics programs at Pwani University and Makerere University, some did not fully commit to the training. One group out six undertaking the mini project in 2020 comprised participants who did not select a preferred project as required and ended up being assigned the remaining project, indicating a lack of commitment to the program. This group did not achieve the project objectives, as participants did not consistently attend training, in contrast with the previous year, when all participants had undergone a competitive selection process. All attendees in subsequent years were from Pwani and Makerere University, and the project phase was in person. Therefore, having a diverse background among the trainee level derails the implementation, forcing the trainers to recap introductory content to disadvantage those in advanced stages and vice versa.

We have success stories from the program, including virtual training. Five members of the 2019 iteration established the Bioinformatics Hub of Kenya Initiative (BHKi) [27] with advisory, mentorship, and support from EANBiT management. This dynamic group is involved in developing a community of students and early-career researchers interested in bioinformatics through online workshops, meetups, seminars, and networking. The organization is fully registered and has attracted grants for its activities. This output demonstrates the impact of RT beyond the duration of training. Participants have continued to form close collaboration and working relationships after training, including manuscripts [28].

## Conclusion

Through EANBiT-RT, we have demonstrated and collected evidence of the benefit of project-based learning in improving skill acquisition and real-world soft skills, including communication, time management, and collaboration. We also show that it is possible to deliver RT using virtual space and reap the benefits of immersive learning with the right tools. But there were challenges, including poor internet connection and the cost of data bundles, which reduced efficiency. Some trainers also struggled with talking to the screen without seeing the participants, walking around the class, helping with technical issues, and effectively gauging the learning experience. The pedagogy of online training is different, and most trainers have not been trained. Programs such as The Carpentries [29] Instructor Training offer these skills [30], and we recommend that trainers be exposed to these concepts to improve virtual training delivery. Although forced by the pandemic this time, online training is expected to become more prevalent; thus, we must adapt to the change. It is necessary to redesign and adjust the curriculum, content, evaluation, and pedagogy to meet virtual training needs.

### Supplementary Information

**Additional file 1.** Moving residential training online. Additional file describes the tools and platforms agreed upon for moving the EANBiT residential training online. It captures three critical issues for the training: video conferencing, collaboration, and resource sharing.

**Additional file 2.** Sample pre-wokshop survey.

**Additional file 3.** Sample post-wokshop survey.

**Additional file 4.** Sample weekly workshop survey.

## Data Availability

Additional material is in Additional file 1 and Sample forms are included in Additional files 2, 3 and 4. The training resources are available from https://github.com/eanbit-rt.
