# Supplementary material for: Designing and delivering bioinformatics project-based learning in East Africa
Source: BMC Bioinformatics. 2024 Apr 14;25:150. doi: 10.1186/s12859-024-05680-2 (PMC11017571; doi:10.1186/s12859-024-05680-2)
Supplement: Supplementary file 1 — Additional file 1. Moving residential training online. Additional file describes the tools and platforms agreed upon for moving the EANBiT residential training online. It captures three critical issues for the training: video conferencing, collaboration, and resource sharing. [file 12859_2024_5680_MOESM1_ESM.pdf]

Additional File 1

Moving EANBiT Residential Training Online

This document details the tools and platforms agreed upon for moving the EANBiT residential training online. We capture three critical issues for the training: video conferencing, collaboration, and resource sharing. For each of the needs, we propose the most preferred tool or platform, citing reasons for its choice and features that can be utilized by the different user groups, i.e. trainers and students.

For the 2020 EANBiT residential training, we will use a combination of tools: Zoom for live training, EANBiT GitHub for content management and collaboration, and Slack for discussions, Q&A, and long-term interactions.

Video Conferencing

Zoom Meetings was the agreed platform to conduct live classes, pieces of training and seminars. The pro package, which starts from USD 15 per month per host, will be used. EANBiT has access to a pro account, which will be used for the training.

Reasons for Choosing Zoom Meetings

1. **Ease of use:** Once installed by the user, the process of creating meetings, joining meetings, and participating in meetings is straightforward. The users do not require high technical skills to use Zoom effectively. Also, its popularity has made it a common choice for many meetings meaning that more people are familiar with it.

2. **Great design and development:** Zoom meetings is a well designed and developed platform with useful features depending on the package of choice. It is also mature for use across different platforms, i.e. Windows OS, Linux OS, Mac OS, Android and iOS. This makes Zoom work well across all these platforms without any functional differences, which is ideal for end-users.

3. **Great Access Control:** Order is a prerequisite to a productive meeting. With a well-implemented access control feature, Zoom Meetings allow for a defined group of users to control the meeting right from its creation through the meeting to the end. Features like waiting rooms improve security by allowing only known attendees to join the meeting, screen sharing restrictions, and muting and unmuting participants by the host. These features, among others, ensure a smooth meeting or training.

Useful Features

Meeting Scheduling

Hosts can schedule meetings and send calendar updates and/or emails to the concerned participants. This feature will allow the calendar and meeting rooms for the different modules to be set in advance and the calendar shared in advance with the trainers and participants. The host also has excellent control of the meeting while creating it; like enforcing a waiting room for all participants, enforcing a password requirement for a meeting, muting participants on entry, enforcing no join before host, etc. Such features allow for better control of the meeting by the host.

In-Meeting Features

I. File Transfer

Zoom allows for in-meeting file transfers with a limit file size of 512MB, allowing the instructor and the students to share materials during the live training. To make use of this feature, click on '**chat**' and then '**file**' to select a file to share from your computer.

II. Screen Sharing

Zoom allows participants to share their screens, making it easier for participants to participate in demos and presentations from their machines. Select the green Share Screen button then you can choose on the screen to share: a presentation, browser, or whiteboard (see below).

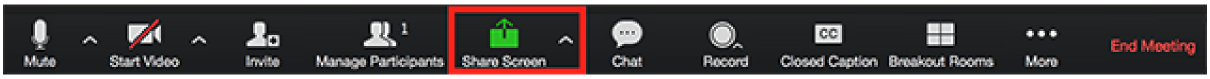

III. White Board

To create a classroom experience, allow for note-taking and scribbling, Zoom provides access to the whiteboard from the Share screen options. The whiteboard can be saved as png. For the best experience, you can use a stylus or a touch screen. Zoom also allows you to annotate over your slides.

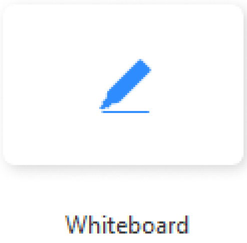

IV. Chats

Participants can chat with one another during the training. This feature can be useful if participants want some help or clarification on some point, which they can get without necessarily disrupting the meeting or leaving the platform.

V. **Meeting Recordings**

Zoom allows the host to record the meeting for future reference if needed. The process is simple and only requires the host to click the **record** button, then zoom will take care of the rest. At the end of the meeting, Zoom saves meeting recordings in different formats; audio files (mp3, m4a, m3u) and video files (mp4).

VI. Breakout rooms

Zoom allows meeting organizers to create independent rooms. These can be used for group discussions or by teaching assistants to support smaller demos.

Contact Groups

Zoom allows one to create contacts and create a user group depending on the goals of the group. As such, group management is left to the Admin/ host, and zoom offers helpful features to support the admin. This feature is great for a classroom setup where students can be placed in contact groups for independent discussions. Contact groups can be added to a channel, which allows for quick meeting invites and chats that last beyond the meeting.

Communication, Discussions, and Collaboration

Slack was the agreed platform for student-teacher, student-student, and group collaboration.

Reasons for Choosing Slack

1. **Ease of use:** Slack uses the idea of a workspace to allow for collaborations. A workspace is created, and users are invited via email or link. Once onboard, they can decide what channels to join depending on their need. While on a channel, they can communicate with other users, and their chats will persist even after log-out.

2. **Training Design:** The content of residential training informed our choice of Slack for collaboration. Students will spend a considerable amount of time doing a mini-project and also have other technical modules to tackle during the period of the training. Slack provides one of the best platforms for diverse collaborations, mainly by development teams for resolving technical issues.

Useful Features

Channels

This feature allows the workspace owner to create a separate place where a team can share messages, tools, and files. Channels can also be used for broadcasting information e.g. An announcement channel whereby only designated people can post but messages are received by all other people in the workspace. Special channels can be created to allow certain users to interact with one another in the form of a group set-up.

Direct Messages

This feature is perfect for conversations that don't require an entire channel to weigh in. Peer-to-peer communications can be done through direct messages.

Mentions

For quicker responses from team members, one can mention a specific member starting with the **@** symbol. A mention ensures the particular mentioned member is immediately notified.

Note Taking

Slack has a built-in note-taking option called posts. Posts enable team members to share and work together on items too big for messages. This is like a text document that team members can pass back and forth, like a simplified Google Documents.

Audio and video calls

Slack supports audio and video calls with up to 15 people. This feature is useful for after-class follow-ups among students or students and trainers.

Content Management and Collaboration

GitHub was the agreed platform for sharing course material and content. Assignments, especially coding assignments and group projects, will be done from this platform.

Reasons for choosing GitHub

1. **Content Management:** GitHub provides a well-documented and straightforward way of managing content. Repositories are used for managing content, and this allows for changes to be made transparently. Also, the visibility of the repositories holding the content can be restricted to particular users.

2. **Training Design:** The residential training has a mini-project and other technical modules. Most technical work and coding is best managed by git, a version control system.

Useful Features

Collaboration

Team members can easily collaborate on a coding project. Features offered by GitHub allows for seamless code reviews by team members making working on a single project by multiple users easier. Some of these features are;

I. **Reviewers**: A student can request for peers to review a section of code or a push request.

II. **Customised comment**: Reviewers can comment on a specific line in a specific file which makes the comment to have a better context thus well understood.

III. **Work branching**: To ensure work is not derailed or new features are implemented simultaneously, branches are provided for this purpose. So students can be working on the same project but each handling a specific feature in their branch. This can also make teachers work easier where they can assess students depending on the work they are doing on their branches.

Cost Implications

I. Using Zoom for Video Conferencing

Bandwidth requirements: Zoom recommends 1.2Mbps for upload and download of HD videos. Most ISPs provide on average speeds of between 9Mbps, download speed to 14Mbps for upload speed within Nairobi city. These speeds will differ from location to location, but on average, users of most telcos will get good speeds across the country with the best connectivity from Safaricom Ltd, Faiba, Airtel, and Telcom. If one is unsure of their connectivity, they can use these tools to check if they will have any issues participating in meetings;

a. Speed Test Tools - Customised for Zoom meetings.
b. Zoom Test Meeting - Good for testing how your internet would perform in case of a real meeting. It also gives you a chance to interact with the Zoom platform if you need to familiarize yourself with the available tools and features Zoom offers in isolation i.e. not in a real meeting environment.

Data Usage: Zoom group meetings consume data as shown below.

Quality	Download	Upload	Total
High	450MB per hour	360MB per hour	810MB per hour
720p	675MB per hour	675MB per hour	1.35GB per hour
1080p	1.2GB per hour	1.2GB per hour	2.4GB per hour

By default, Zoom uses the minimum available bandwidth. Also, HD video streaming is not enabled; most video calls will be between High Quality - 720p. Thus, the average data usage per hour would range between 810MB - 1.35GB. The usage may increase or decrease

depending on how one uses the platform. Data usage may increase if one records a session with an additional 50MB per hour for voice recording and more for video recording. NB: Data usage may increase with the number of participants in the meeting, mostly because you might need to download more data compared to a smaller group. Here are some great tips to reduce data usage.

For a 4 hour day, when using Zoom, an average of 3.2GB - 5.4GB will be required. Below are the various charges from the different telcos, mentioned above, that would suit this need;

a. **Safaricom**
   i. Buy 3-5 1GB daily bundle each at 99 Bob. Total: KES. 297 - 495

b. **Faiba**
   i. Buy 3-5 1GB daily bundle each at 50 Bob. Total: KES. 150 - 250
   ii. Buy 1 8GB weekly bundle at 300Bob. This will spare up to 3GB for next day's use or so.

NB: Faiba may have a limitation i.e. A few phones can support it. Read more here.

c. **Airtel**
   i. Buy 2 2GB daily bundle each at 99Bob. Total: KES. 198

d. **Telcom**
   i. Buy 2 2GB daily bundle each at 100Bob. Total: KES. 200

These prices are not fixed and will vary depending on the package selected.

II. Using Slack and Git

These platforms do not consume as much data. Slack will mostly be used for collaboration using text as the main mode of communication. Text is usually the simplest data format to exchange and does not require high bandwidth or consume lots of data. Only files may consume more data depending on the type, content and size.

Git, on the other hand, consumes as much data as the content being shared. Our primary use for GitHub is content management especially Project collaborations, there are a few things that could be done to keep projects as small as possible.

1. Only track changes to your project and avoid, by all means, coupling the project and its environment together. Environments are usually very big yet are not special to a program, they can be recreated by other parties when guided how to do so.

2. Untrack files that are IDE specific. Some IDEs will create .idea files which details some specifics of the project while using that IDE. These are not necessary since different people might have different preferences on the IDE of choice.
