# Supplementary material for: Designing and delivering bioinformatics project-based learning in East Africa
Source: BMC Bioinformatics. 2024 Apr 14;25:150. doi: 10.1186/s12859-024-05680-2 (PMC11017571; doi:10.1186/s12859-024-05680-2)
Supplement: Supplementary file 2 — Additional file 2. Sample pre-wokshop survey. [file 12859_2024_5680_MOESM2_ESM.pdf]

Additional File 2

EANBiT Residential Training - 2022

The Eastern Africa Network for Bioinformatics Training (EANBiT) is conducting an intensive bioinformatics course online and in-person RT in July 2022.

Kindly complete this pre-workshop survey to assist the trainers in tailoring the course to your needs.

* Indicates required question

Personal details

1. Name *

2. Surname *

3. Age Range *

Mark only one oval.

- ( ) 20-24
- ( ) 25-29
- ( ) Over 30

4. Gender *

Mark only one oval.

- Female
- Male
- Prefer not to say
- Other: ____________________

5. Email address *

____

6. Main citizenship *

____

Affiliation

This section is about your current institution.

7. What is the name of your sponsoring program? *

Mark only one oval.

- EANBiT
- BRecA
- Self
- Other: ____________________

8. University

Mark only one oval.

- Pwani University
- Makerere University

Training Needs and proposed courses

In this section, we would like to gauge your familiarity with various aspects of the topics covered in the EANBiT training. Please answer the questions honestly.

9. How comfortable are you with the Linux commandline?

Mark only one oval.

	1	2	3	4	5	
Nev						Advanced

10. How comfortable are you with tidyverse?

Mark only one oval.

	1	2	3	4	5	
Nev						Expert level

11. How comfortable are you with R-markdown?

Mark only one oval.

	1	2	3	4	5	
Neve	◯	◯	◯	◯	◯	Advanced

12. How comfortable are you with Git and GitHub?

Mark only one oval.

	1	2	3	4	5	
Neve	◯	◯	◯	◯	◯	I can fix merge conflicts like a pro

13. How comfortable are you with workflow languages?

Mark only one oval.

	1	2	3	4	5	
Neve	◯	◯	◯	◯	◯	Can use 3 or more

14. Which workflow languages are you comfortable with?

Check all that apply.

- [ ] Nextflow
- [ ] Snakemake
- [ ] Galaxy Project
- [ ] Other: ______________________

15. Please comment on your choices on the 'how comfortable are you' questions above.

16. How familiar are you with Containers: Docker, Singularity?

Mark only one oval.

	1	2	3	4	5	
Nev	◯	◯	◯	◯	◯	Have used both of them

17. Have you analysed long-read sequence data (PacBio, ONT)?

Mark only one oval.

◯ Yes

◯ No

18. Please comment on you exposure to and experience with long-read sequence data (PacBio, ONT)

Read this article for some information https://bmcbioinformatics.biomedcentral.com/articles/10.1186/s12859-018-2446-1

Your motivation for this training

This section is about what brings you to attend the EANBiT training

19. What are the skills you want to strengthen by attending this training? *

Thank You

This content is neither created nor endorsed by Google.

Google Forms
