# Supplementary material for: Designing and delivering bioinformatics project-based learning in East Africa
Source: BMC Bioinformatics. 2024 Apr 14;25:150. doi: 10.1186/s12859-024-05680-2 (PMC11017571; doi:10.1186/s12859-024-05680-2)
Supplement: Supplementary file 3 — Additional file 3. Sample post-wokshop survey. [file 12859_2024_5680_MOESM3_ESM.pdf]

Additional File 3

EANBiT Residential Training Post-Training Survey

Thank you for your engaging participation in this Year's Residential Training. We would appreciate your feedback. Kindly take a few minutes to fill in this form.

* Indicates required question

Personal details

1. Age Range *

Mark only one oval.

- ( ) 20-24
- ( ) 25-29
- ( ) Over 30

2. Gender *

Mark only one oval.

- ( ) Female
- ( ) Male
- ( ) Prefer not to say

3. Main citizenship *

____

Competencies acquired from the Modules

Having gone through the training, please update your familiarity with various aspects of the topics covered in the EANBiT training. This feedback will allow us to determine the impact of the training on your skills.

4. Have your research interests changed/ evolved during this training? What are your research interests? *

____
____
____
____
____

5. How comfortable are you with the Linux commandline? *

Mark only one oval.

	1	2	3	4	5	
Nev	◯	◯	◯	◯	◯	Advanced

6. How comfortable are you with tidyverse? *

Mark only one oval.

	1	2	3	4	5	
Nev	◯	◯	◯	◯	◯	Expert Level

7. How comfortable are you with Git and GitHub? *

Mark only one oval.

	1	2	3	4	5	
Nev	◯	◯	◯	◯	◯	I can fix merge conflicts like a pro

8. How comfortable are you with Containers: Docker, Singularity? *

Mark only one oval.

	1	2	3	4	5	
Bare	◯	◯	◯	◯	◯	Very Comfortable

9. How comfortable are you with workflow languages? *

Mark only one oval.

	1	2	3	4	5	
Nev	◯	◯	◯	◯	◯	Can use 3 or more

10. Please comment on how your familiarity of the above tools has changed and influenced by the training. *

____

____

____

____

____

How would you Rate the Modules

In this section, we seek for overall experience you had with various modules. Please honestly rate (5-excellent) the module based on content, delivery, accessibility, and impact.

11. Reproducibility: Containers, Workflows, Git and GitHub *

Mark only one oval.

1	2	3	4	5
◯	◯	◯	◯	◯

12. Advanced Linux and R *

Mark only one oval.

1	2	3	4	5
◯	◯	◯	◯	◯

13. Whole Genome and Metagenomics *

Mark only one oval.

1	2	3	4	5
◯	◯	◯	◯	◯

14. Long Read Sequencing *

Mark only one oval.

1	2	3	4	5
◯	◯	◯	◯	◯

15. Ethics Workshop *

Mark only one oval.

1	2	3	4	5
◯	◯	◯	◯	◯

16. Proposal Writing Sessions *

Mark only one oval.

1	2	3	4	5
◯	◯	◯	◯	◯

17. Reference Management with Mendeley *

Mark only one oval.

1	2	3	4	5
◯	◯	◯	◯	◯

18. Use this section to comment on any of your ratings above.

The Training Experience

This section will help us hear from you on the entire training experience.

19. Topics covered were relevant to me. *

Mark only one oval.

- Strongly disagree
- Disagree
- Neutral
- Agree
- Strongly agree

20. Content was organized and easy to follow. *

Mark only one oval.

- Strongly disagree
- Disagree
- Neutral
- Agree
- Strongly Agree

21. Materials distributed were helpful. *

Mark only one oval.

- Strongly disagree
- Disagree
- Neutral
- Agree
- Strongly Agree

22. The time allocated for the modules was sufficient. *

Mark only one oval.

- Strongly disagree
- Disagree
- Neutral
- Agree
- Strongly agree

23. What aspects of the training did you enjoy and benefit from the most? (Briefly explain) *

24. What are some challenges that you faced while participating in the training? *

25. Given your experience as a participant, if you were allowed to organize for this training, what would you do differently? *

26. Do you have any other comment? (Briefly state) *

Thank You!

It was great having you participate in this training and we appreciate your feedback as well. We wish you the very best moving forward!

This content is neither created nor endorsed by Google.

Google Forms
