# Supplementary material for: Designing and delivering bioinformatics project-based learning in East Africa
Source: BMC Bioinformatics. 2024 Apr 14;25:150. doi: 10.1186/s12859-024-05680-2 (PMC11017571; doi:10.1186/s12859-024-05680-2)
Supplement: Supplementary file 4 — Additional file 4. Sample weekly workshop survey. [file 12859_2024_5680_MOESM4_ESM.pdf]

Additional File 4

EANBiT RESIDENTIAL TRAINING COURSE

FEEDBACK FORMS: WEEK

Kindly select either 1,2,3,4 or 5 in the table below to respond to the next questions where:
1 is Strongly Agree **2** is Agree **3** is Neutral
4 is Disagree **5** is Strongly Disagree

No		1	2	3	4	5
01.	The objectives of the training were clearly defined					
02.	Participation and interaction were encouraged					
03.	Topics covered were relevant to me					
04.	Content was organized and easy to follow					
05.	Materials distributed were helpful					
06.	Training experience so far will be useful for my work					
07.	Trainers were knowledgeable on training topics					
08.	Trainers were well prepared					
09.	The time allocated for the modules was sufficient					
10.	The meeting room and facilities were adequate and comfortable					

11. What did you like most about this week of training?

----

12. What aspects of the training could be improved?

----

13. How do you hope to change your practice as a result of the training so far?

----

14. How have the food and accommodation services been?

